# A261 INVESTIGATING THE RELATIONSHIP BETWEEN AIR POLLUTION AND BIOMARKERS OF CROHN’S DISEASE IN CANADA

**DOI:** 10.1093/jcag/gwad061.261

**Published:** 2024-02-14

**Authors:** J SHAO, M Xue, A Neustaeter, S Lee, H Leibovitzh, E I Benchimol, W Turpin, K Croitoru

**Affiliations:** University of Toronto, Toronto, ON, Canada; Lunenfeld-Tanenbaum Research Institute, Toronto, ON, Canada; Lunenfeld-Tanenbaum Research Institute, Toronto, ON, Canada; Lunenfeld-Tanenbaum Research Institute, Toronto, ON, Canada; Lunenfeld-Tanenbaum Research Institute, Toronto, ON, Canada; University of Toronto, Toronto, ON, Canada; Lunenfeld-Tanenbaum Research Institute, Toronto, ON, Canada; Lunenfeld-Tanenbaum Research Institute, Toronto, ON, Canada

## Abstract

**Background:**

The cause of Crohn's disease (CD) remains unclear. Studies suggest a potential connection between CD and air pollution, with a higher occurrence of CD found in areas with higher pollution levels.

**Aims:**

Our study sought to explore this potential connection by investigating the relationship between components of air pollution and biomarkers indicative of CD risk.

**Methods:**

In total, 2,256 healthy first-degree relatives of patients with CD were recruited as part of the CCC-GEM Project. We assessed baseline samples of gut permeability using the urinary fractional excretion ratio of lactulose-to-mannitol (LMR), subclinical inflammation via fecal calprotectin (FCP), and stool microbiome using 16S-rRNA sequencing. Air pollutant (n=8) concentrations were obtained from the National Air Pollution Surveillance Database. We used postal code to estimate air pollutant exposure via inverse distance weighting, averaging data for one (pollutant_1-month_), three (pollutant_3-months_), and twelve months (pollutant_12-months_) prior to recruitment. We used generalized estimating equation models to find associations between pollutant exposure and LMR, FCP, and microbiome composition, adjusting for age, sex, air pollutant seasonality, familial income, and familial relationships. Significance was set at pampersand:003C0.05, and false discovery rate (q) correction was applied to microbiome analysis.

**Results:**

We observed a negative association between particulate matter (PM_µg/m_^3^), PM10_1-month_, and PM10_3-months_ and LMR (β=-0.06[-0.09, -0.01], p=6.0×10^-3^ and β=-0.05[-0.07, -0.01], p=7.6×10^-4^), and a positive association between PM10_3-months_ and FCP (β=0.05[0.001, 0.10], p=0.04). 12-month pollutant exposure was associated with seven microbial taxa in a logit model. Notably, Carbon monoxide (CO)_12-months_ was associated with Oscillospiraceae UCG_003 (β=3.49[1.53, 5.46], q=0.03). PM25_12-months_ was associated with Marvinbryantia (β=0.12[0.06, 0.18], q=0.02). PM10_12-months_ was associated with Moryella (β=0.07[0.03, 0.10], q=0.02). Nitrogen dioxide (NO)_12-months_ was associated with Coprobacter and Ruminococcaceae UBA1819 (β=0.05[0.02, 0.07], q=0.03 and β=0.05[0.02, 0.08], q=0.04). NOX_12-months_ was also associated with Ruminococcaceae_UBA1819 as well as Intestinimonas (β=0.03[0.01, 0.04], q=0.04 and β=0.03[0.01, 0.04], q=0.02).

**Conclusions:**

Air pollutant exposure was associated with biomarkers of gut inflammation, barrier function, and microbiome composition. A shorter exposure (1/3 months) primarily affected FCP and LMR, while longer-term exposure (12 months) exerted a discernible impact on the microbiome. These findings suggest that exposure to air pollution may modulate the gut barrier function, inflammation, and the gut microbiome, biomarkers that are associated with the development of CD.

**Funding Agencies:**

Crohn’s and Colitis Canada Genetics Environment Microbial (CCC-GEM) III, Leona M. and Harry B. Helmsley Charitable Trust, Dr. Croitoru is the recipient of the Canada Research Chair in Inflammatory Bowel Diseases

